# Dihydroquercetin Activates AMPK/Nrf2/HO-1 Signaling in Macrophages and Attenuates Inflammation in LPS-Induced Endotoxemic Mice

**DOI:** 10.3389/fphar.2020.00662

**Published:** 2020-05-19

**Authors:** Liming Lei, Yunfei Chai, Haoming Lin, Chunbo Chen, Mingyi Zhao, Weiping Xiong, Jian Zhuang, Xiaoping Fan

**Affiliations:** ^1^ Department of Intensive Care Unit of Cardiovascular Surgery, Guangdong Cardiovascular Institute, Guangdong Provincial People’s Hospital, Guangdong Academy of Medical Sciences, Laboratory of South China Structural Heart Disease, Guangzhou, China; ^2^ Department of Anesthesiology of Guangdong Cardiovascular Institute, Guangdong Provincial People’s Hospital, Guangdong Academy of Medical Sciences, Guangzhou, China; ^3^ Department of Hepatobiliary Pancreatic Surgery, Sun Yat-sen Memorial Hospital, Sun Yat-sen University, Guangzhou, China; ^4^ Department of Pediatrics, the Third Xiangya Hospital, Central South University, Changsha, China; ^5^ Department of Cardiovascular Surgery of Guangdong Cardiovascular Institute, Guangdong Provincial People’s Hospital, Guangdong Academy of Medical Sciences, Laboratory of South China Structural Heart Disease, Guangzhou, China

**Keywords:** dihydroquercetin, endotoxemia, AMP-activated protein kinase, nuclear factor erythroid 2-related factor 2, heme oxygenase-1, anti-inflammation

## Abstract

Dihydroquercetin (DHQ) is a flavonoid compound known for its anti-oxidant effects. Oxidative stress plays a dominant role in regulating the pathways associated with systemic inflammatory immune activation during endotoxemia. Whether and how DHQ regulates inflammatory responses in endotoxemia remains elusive. Here we show DHQ pretreatment effectively reduced the Ten-day mortality in bacterial endotoxin lipopolyssacharide (LPS)-challenged mice, suppressing LPS-induced inflammatory responses reflected by impaired production of tumor necrosis factor α (TNF-α) and interleukin-6 (IL-6) in the serum of mice. In Raw 264.7 cells, DHQ pretreatment significantly inhibited the transcriptional upregulation of TNF-α, interferon-γ (IFN-γ), interleukin-10 (IL-10) and toll-like receptor 4 (TLR-4) after LPS stimulation. Additionally, knockdown of heme oxygenase-1 (HO-1), one of the most important DHQ induced antioxidant genes, cancelled the inhibition of DHQ treatment on LPS induced TNF-α, IFN-γ production. Nuclear factor erythroid 2-related factor 2 (Nrf2) expression and AMP-activated protein kinase (AMPK) phosphorylation were both enhanced by DHQ in Raw 264.7 cells, indicating a DHQ induced AMPK/Nrf2/HO-1 signal axis. In conclusion, DHQ pretreatment could protect mice against the inflammation and mortality associated with endotoxemia.

## Highlights

1. Dihydroquercetin attenuates inflammation in LPS-induced endotoxemic mice.
2. Dihydroquercetin reduces inflammatory response in RAW264.7 cells.
3. Nrf2/HO-1 mediates the anti-inflammatory effect of Dihydroquercetin.

## Introduction

Endotoxemia is involved in a wide range of fatal diseases, such as hepatitis, acute pancreatitis, pneumonia, and trauma. Acute endotoxemia elicits a cascade of pathophysiological changes involving multiple organs, and systems, ultimately leading to infectious shock and multiple organ failure. Pathogenesis of endotoxemia is associated with the hyper-activation of the innate immune cells and the spontaneous secretion of systemic inflammatory cytokines in response to the stress induced by microbial infections (Gawish et al., 2015; Inoue and Shinohara, 2015; Maitre et al., 2015). Macrophages are professional phagocytic cells that play critical roles in host innate immunity, producing a large number of pro-inflammatory cytokines including tumor necrosis factor-α (TNF-α) and interleukin-6 (IL-6), providing a first-line defense against intracellular pathogens (Costa et al., 2014; Zullo et al., 2015). Macrophage dysfunction has been implicated in tissue dysfunction and endotoxic mortality during endotoxemia (Kambara et al., 2015; Park et al., 2015; Xu et al., 2015). Therefore, targeting deregulation of immune cells, such as macrophages, is a potential strategy for the prevention or amelioration of deleterious outcomes in endotoxemia.

Dihydroquercetin (DHQ; 3, 5, 7, 3′, 4′-pentahydroxy-flavanone) (Oi et al., 2012) is an operative flavonoid, abundantly found in olive oil, grapes, in citrus fruits, and onions with molecular weight of 304.25 (**Figure S1**) (Akinmoladun et al., 2018). It is water-alcohol and polar solvents soluble. There are two forms of DHQ, the trans- and cis-form. The trans-DHQ oxidizes more actively, providing hydrogen atoms to form the oxidation product quercetin [2-(3,4-dihydroxyphenyl)-3,5,7-trihydroxy-4H-chromen-4- one] (Sunil and Xu, 2019). DHQ shows a tremendous variety of pharmacological and biochemical consequences, including hepatoprotective, anti-diabetic, cardioprotective, antitumor, and aneuroprotective effects (Oi et al., 2012). It attenuates oxidative stress by upregulating Nuclear factor erythroid 2-related factor 2 (Nrf2)-associated antioxidant genes, including Heme oxygenase-1 (HO-1), NAD(P)H quinone oxidoreductase-1 (NQO1), and glutamate-cysteine ligase modifier subunits (Zhao et al., 2015). We recently reported that administration of DHQ could inhibit oxidative stress in Concanavalin A (ConA)-induced liver injury *via* upregulation of HO-1 through the AMPK/Nrf2 signaling pathway in macrophages/Kupffer cell (Zhao et al., 2015). This finding indicates that Nrf2 and HO-1 were both involved in the mechanism by which DHQ protects against oxidative stress. However, to the best of our knowledge, the anti-inflammatory effects of DHQ have not been thoroughly investigated.

Nrf2 serves as one of the key regulators of cellular defense against oxidative stress. Upon exposure to oxidative stress, Nrf2 translocates into the nucleus, where it binds to cis-acting antioxidant-responsive elements (AREs) and promotes the transcription of a broad range of cytoprotective genes, including HO-1 (Krajka-Kuzniak et al., 2017). HO-1 is an essential enzyme that degrades heme to bilirubin, carbon monoxide (CO) and iron (Dunn et al., 2014). Endogenous CO inhibits the expression of LPS-induced pro-inflammatory cytokines and increases LPS-induced expression of interleukin-10 (IL-10) in macrophages, suggesting the anti-inflammatory effect of HO-1 (Otterbein et al., 2000). Moreover, recent studies have shown that inflammatory challenges could promote HO-1 expression, which could blunt expression of pro-inflammatory cytokines such as TNF-α, and limit inflammatory injuries. Administration of a HO inhibitor significantly suppressed the inhibitory effects of IL-10 on LPS-induced TNF-α and nitric oxide (NO) production in macrophages (Lee and Chau, 2002). These studies suggest antioxidant Nrf2/HO1 pathway could limit over-activated immune responses. Hence, it is necessary to identify whether DHQ holds the potential as a novel anti-inflammatory drug in endotoxemia.

In this study, we aimed to explore the potential effects of DHQ on the acute inflammatory responses by administering it to LPS-challenged endotoxemic mice and Raw264.7 macrophage cells. Our study provided evidence of a protective role for DHQ in LPS-induced endotoxemic mice, reducing the production of pro-inflammatory cytokines. These results demonstrate the critical anti-inflammatory role of Nrf2/HO-1 in macrophages, and should help facilitate the development of therapeutic strategies for the control of inflammatory diseases.

## Materials and Methods

### Animals and Treatments

**Figure 1:**
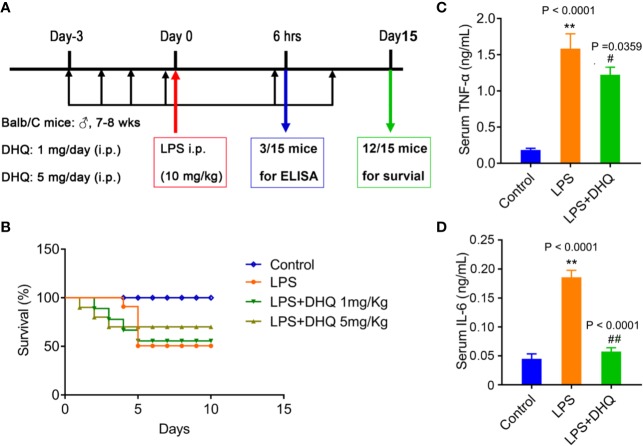
DHQ reduced mortality and serum inflammatory cytokines in endotoxemic mice. **(A)** Schematic illustration of the induction of endotoxemia and treatment modality in mice (n=12). **(B)** Survival of mice after induction of endotoxemia. **(C)** Serum TNF-α levels in endotoxemia mice, *P* < 0.0001 in one-way ANOVA test, *P* value of Tukey's *post hoc* test between groups were presented within panels. **(D)** Serum IL-6 levels in endotoxemia mice, *P* < 0.0001 in one-way ANOVA test. Data are means ± SD. **P < 0.01 vs. control group. ^#^ *P* < 0.05, ^##^ *P* < 0.01 vs. LPS group.

All the animals were authorized by Research Ethics Committee of Guangdong Provincial People's Hospital (license GDREC2019103A). Sixty male Balb/c mice (20–25g, 7–8 weeks) were purchased from Cavens Experimental Animals Company of Changzhou and housed in the Guangdong Provincial People's Hospital Experimental Animal Center. All 60 mice were kept in individual cages under standard conditions (12 h light and 12 h dark cycle). Animal care and experiments were performed in accordance with the Animal Research: Reporting of In Vivo Experiments (ARRIVE) guidelines (Kilkenny et al., 2010). The 60 mice were equally divided into four groups: the control group (n=15), the endotoxemic model group (n=15), and two treatment groups (n=15) administered low (1 mg/kg) and high (5 mg/kg) doses of Dihydroquercetin (DHQ, Beckmann-Kenko GmbH, Germany), respectively, once daily. While the control group was treated with equal volume normal saline solution, the mice in the treatment groups received DHQ by intraperitoneal (i.p.) injection for 3 days before the induction of endotoxemia. To induce endotoxemia, mice were challenged with an i.p. injection of LPS (L6529, Sigma-Aldrich, USA) (10 mg/kg). Three mice from each group were sacrificed 6 h after LPS challenge, blood was collected. The remaining 12 mice in each group were monitored every 12 h for up to 10 days and mortality rate was recorded. The schematic illustration of the animal experiment was provided in **Figure 1A**. This study was carried out in strict accordance with the recommendations in the Guide for the Care and Use of Laboratory Animals of the National Institutes of Health. The animal use protocol has been reviewed and approved by the Institutional Animal Care and Use Committee (IACUC) of the Guangdong Provincial People's Hospital.

### Enzyme Linked Immunosorbent Assay (ELISA)

Blood collected from mice was allowed to clot at room temperature (RT), and then centrifuged (5804R, Eppendorf, USA) for 15 min at 1,500 g to separate the serum. The serum samples were transferred into another tube and stored at -20°C before analysis. Serum levels of TNF-α and IL-6 were measured using ELISA with commercially available kits (Thermo Fisher Scientific Inc., USA), according to the manufacturer's instructions. Each sample was tested in duplicate.

### Cell Culture and Treatment

Raw264.7 cells, a macrophage-like cell line, were purchased from the American Type Culture Collection (Manssas, VA, USA), cultured in Dulbecco's modified Eagle's medium supplemented with 2 mM glutamine (25030081, Thermo Fisher Scientific Inc., USA), penicillin G (100 U/ml, sc-257971B, Santa Cruz, USA), streptomycin (100 μg/ml, 3810-74-0, Gold Biotechnology, USA), and 10% fetal bovine serum (ExCell Bio, Shanghai, China), and maintained at 37°C in a humidified incubator containing 5% CO_2_. To investigate the potential effects of DHQ on LPS-induced inflammatory responses, Raw264.7 cells were pretreated with DHQ (100 μM) for 6 h, followed by addition of LPS to the final concentration of 10 μg/ml. The cells were further incubated for another 24 h before processing. 10 μM AMPK inhibitor (Compound C, CC, B3252, APExBIO, USA) and 20nM si-AMPK (Santa Cruz, CA, USA) pretreatment for 24 h before the LPS stimulation.

### Quantitative Real-Time Polymerase Chain Reaction (PCR)

Total RNAs from Raw264.7 cells were extracted using ISOGEN (Nippon Gene, Tokyo, Japan) following the manufacturer's protocol. RNAs were reverse transcribed to cDNAs using the PrimeScript TR reagent kit (Takara, Japan), using the following primers: TNF-α: Forward: 5—TGT CTA CTG AAC TTC GGG GTG AT—3, Reverse: 5—AAC TGA TGA GAG GGA GGC CAT—3; INF-γ: Forward: 5—CAA GGC GAA AAA GGA TGC A—3, Reverse: 5—CGG ATG AGC TCA TTG AAT GCT—3; IL-10: Forward: GGG TGA GAA GCT GAA GAC CCT, Reverse: TCA CCT GCT CCA CTG CCT T; HO-1: Forward: CAG GGT GAC AGA AGA GGC TAA GAC, Reverse: TTG TGT TCC TCT GTC AGC ATC AC; TLR-4: Forward r: GAG CTT CAA CCC CTT GAA GAT CT, Reverse: CCA TGC CAT GCC TTG TCT T; OPN: Forward: CCC GGT GAA AGT GAC TGA TTC T, Reverse: GAT TCT GCT TCT GAG ATG GGT CA. Real time PCR was performed in a 25 μl reaction system containing 12.5 μl 2× Premix Ex Taq^™^ (Takara, Shiga, Japan), 5 μl of primer, 6.5 μl of cDNA. Amplification was conducted on the Applied Biosystem PRISM7700 (ABI Japan, Co., Ltd., Tokyo, Japan). Quantification was determined by the standard curve and 2^-ΔΔCt^ methods.

### Protein Extraction and Western Blot

Cells were collected and lysed in RIPA buffer with phenylmethanesulfonyl fluoride (PMSF). Cells were disrupted by repeated aspiration by pipette and incubated on ice for 30 min. Samples were transferred into new Eppendorf tubes and centrifuged at 12,000 rpm at 4℃ for 30 min. The supernatant was carefully transferred into new tubes and preserved at -20℃ until further use. The protein concentration was determined using a BCA Protein Assay Kit (Pierce, USA). Proteins of equal quantity were subjected to sodium dodecyl sulfate-polyacrylamide gel electrophoresis and transferred onto polyvinylidene fluoride (PVDF) membranes (Bio-Rad, Hercules, CA). The membranes were blocked with 3% skim milk containing 0.1% sodium azide (Santa Cruz Biotech) for 1 h at RT, then incubated with primary antibodies overnight at 4℃. After thorough washing, membranes were probed with horseradish peroxidase-conjugated secondary antibodies for 1 h at RT. Blots were developed with an enhanced chemiluminescence system. Western blots were scanned and analyzed by a Gel-Pro Analyzer version 3.0.

### Flow Cytometry

Cellular expression of HO-1 was assessed by intracellular staining using the BD cytofix/cytoperm Fixation/Permeabilization Kit according to the manufacturer's instructions, and staining was measured by flow cytometry. Briefly, cells were collected, washed in PBS, fixed with 4% paraformaldehyde and permeabilized with 0.1% saponin. Permeabilized ells were incubated with fluorescein isothiocyanate (FITC)-conjugated anti-HO-1 antibody (Abcam, ab69545) for 20 min.

### Immunofluorescence Assay

Cells grown on coverslips were fixed with 4% paraformaldehyde for 10 min, permeabilized with 0.5% Triton X-100, and blocked with 1% bovine serum albumin (BSA) in PBS for 1 h at RT. For staining of Nrf2, the cells were further incubated with anti-Nrf2 (Santa Cruz, 1:200) antibodies at 4℃ overnight, followed by incubation with a secondary FITC-conjugated antibody for 1 h at RT. Cell nuclei were counterstained with propidium iodide (PI, 0.10 g/ml) for 1 min and mounted for immunofluorescence imaging. Images were obtained under a confocal microscope (Olympus, Tokyo, Japan).

### Statistical Analysis

Data are expressed as means ± SD. Statistical comparisons were determined by one-way ANOVA combined with Tukey's *post hoc* test using SPSS software version 22.0 (SPSS, Chicago, IL, USA). *P* values <0.05 was considered statistically significant.

## Results

### DHQ Pretreatment Reduced the Mortality of Endotoxemic Mice

I.p. administration of large-dose LPS induce endotoxemic shock and death in mice to explore how DHQ treatment affects inflammation, as intraperitoneal injection of LPS is a well-established method used to model endotoxemia. In this study, 5 days after LPS injection, only 41.67% (5/12) of the mice in the model group survived (**Figure 1B**). Meanwhile, the survival rates of mice in the DHQ treatment groups at this time were 58.33% (7/12) (1 mg/kg DHQ) and 75% (9/12) (5 mg/kg DHQ), respectively. After 10 days, the survival rate of the model group remained at 41.67% (5/12). In contrast, the survival rate of mice in the DHQ low-dosage group decreased to 50% (6/12), while no more mice died in DHQ high-dosage group (75% survived). Kaplan-Meier analysis indicated that time to death was significantly shorter in the control group than the DHQ treatment groups, suggesting that DHQ pretreatment could effectively rescue the endotoxic mortality in mice.

### DHQ Pretreatment Attenuated Systemic Inflammation in Endotoxemic Mice

Systemic inflammation disturbance plays a key role in the outcome of endotoxemia. We therefore set out to investigate whether DHQ pretreatment could influenced inflammation, especially inflammatory cytokines secretion during endotoxemia. In model mice 6 h after LPS challenge serum TNF-α (0.185 ± 0.023 ng/ml *v.s.* 1.586 ± 0.203 ng/ml, *P* < 0.0001) and IL-6 (44.9 ± 4.9 pg/ml *v.s.* 185.7 ± 6.9 pg/ml, *P* < 0.0001) levels were significant increased, in line with the induction of acute inflammation. However, pretreatment with DHQ significantly reduced serum levels of both TNF-α (1.586 ± 0.203 ng/ml vs. 1.22 ± 0.11 ng/ml, *P* = 0.0359) and IL-6 (185.7 ± 6.9 pg/ml vs. 57.5 ± 3.8 pg/ml, *P* < 0.0001) (**Figures 1C, D**), indicating that DHQ pretreatment exerted anti-inflammatory effects in endotoxemic mice.

### DHQ Produced Anti-Inflammatory Effects in LPS-Challenged Raw264.7 Cells

**Figure 2:**
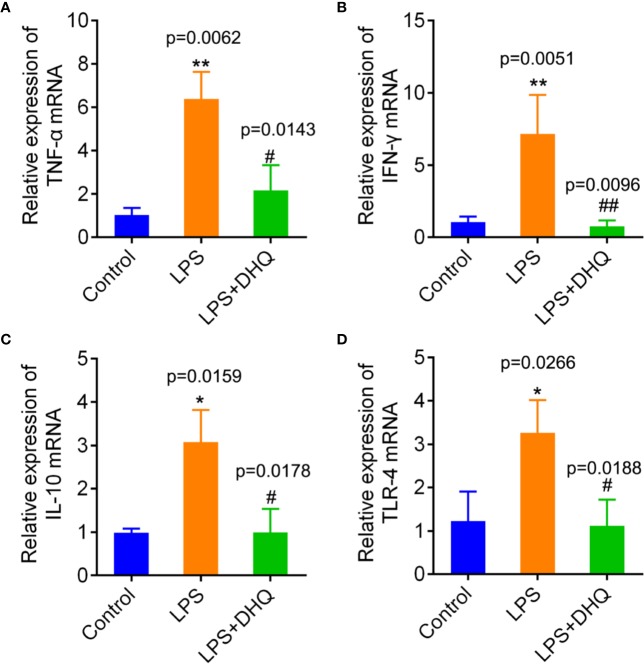
Effects of DHQ on expression of inflammatory genes in cultured Raw264.7 cells after LPS challenge. RT-PCR revealed that DHQ pretreatment significantly reduced the levels of TNF-α (*P* = 0.0054) **(A)**, IFN-γ (*P* = 0.0034) **(B)**, IL-10 (*P* = 0.0106) **(C)**, and TLR-4 (*P* = 0.0131) **(D)** in LPS-challenged groups. *P* value of Tukey's *post hoc* test between groups were presented within panels. *P < 0.05, **P < 0.01 vs. control group. ^#^ *P* < 0.05, ^##^ *P* < 0.01 vs. LPS group.

Macrophages play a regulatory role in many inflammatory responses, so we further examined the direct effects of DHQ on a cultured macrophage cell line, Raw264.7 *in vitro*. RAW264.7 cells were stimulated with 10 μg/ml LPS for 24 h in the presence of DHQ. As shown in **Figure 2**, incubation with LPS induced remarkable transcriptional upregulation of cytokines TNF-α (1.04 ± 0.32 vs. 6.37 ± 1.24, *P* = 0.0062), IFN-γ (1.05 ± 0.38 vs. 7.16 ± 2.68, *P* = 0.0051), IL-10 (0.99 ± 0.09 vs. 3.08 ± 0.74, *P* = 0.0159), and the LPS receptor TLR-4 (1.23 ± 0.68 vs. 3.26 ± 0.76, *P* = 0.0226) in Raw264.7 cells. In comparison, DHQ significantly suppressed expression of TNF-α (6.37 ± 1.24 vs 2.17 ± 1.16, *P* = 0.0143), IFN-γ (7.16 ± 2.68 vs. 0.76 ± 0.41, *P* = 0.0096), IL-10 (3.08 ± 0.74 vs. 1.00 ± 0.53, *P* = 0.0178), and TLR-4 (3.26 ± 0.76 vs. 1.12 ± 0.60, *P* = 0.0188). The western blotting analysis showed the expected expression consistent with the mRNA in **Figure S2**. These results indicate DHQ could also effectively blunt the LPS induced inflammatory response of Raw264.7 cells, reducing the secretion of inflammatory cytokines.

### DHQ Activated AMPK/Nrf2/HO-1 Signaling in Macrophages

**Figure 3:**
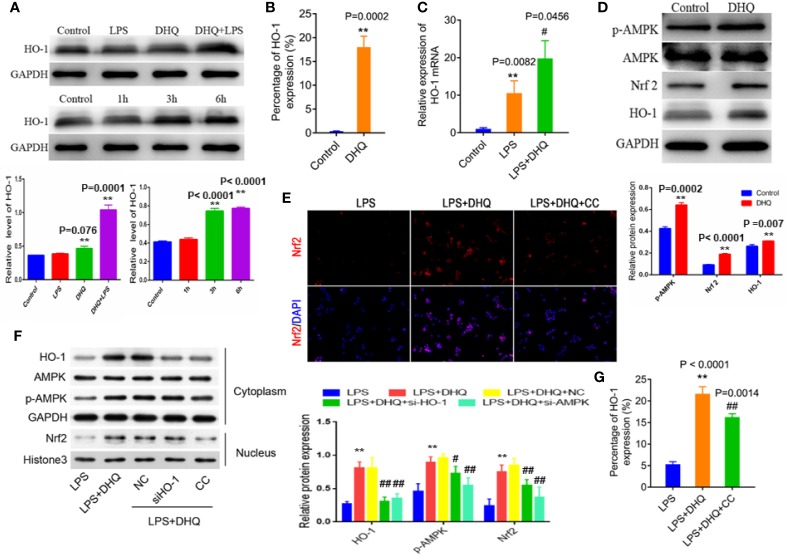
DHQ increases HO-1 expression and activated AMPK/Nrf2/HO-1 signaling in Raw264.7 cells. **(A)** Up panel: Western blotting analysis of HO-1 protein. Down panel: Time-dependent increase in HO-1 induced by DHQ. Bar graphs illustrate the protein expression of HO-1. Values are expressed as mean ± SD. *P* < 0.001 in one-way ANOVA test, **P < 0.01 vs. control group. **(B)** Flow cytometeric analysis of intracellular HO-1, *P* = 0.0002, **P < 0.01 vs. control group. **(C)** RT-PCR analysis of HO-1 mRNA. *P* = 0.0033 in one-way ANOVA test, *P* value of Tukey's *post hoc* test between groups were presented within panels. **P < 0.01 vs. control group. ^#^ *P* < 0.01 vs. LPS group. **(D)** Western blotting analysis of p-AMPK, AMPK, Nrf2, and HO-1 in macrophages. Bar graphs illustrate the protein expression of p-AMPK, Nrf2, HO-1. Values are expressed as mean ± SD, **P < 0.01 vs. control group. **(E)** Immunofluorescent staining of Nrf2 in macrophages increased after DHQ or CC treatment. **(F)** Western blotting analysis of the indicated proteins in the cytoplasm and nucleus of RAW264.7 cells with indicated treatment. Bar graphs illustrate the protein expression of HO-1, p-AMPK, Nrf2. Values are expressed as mean ± SD. *P* < 0.0001 in one-way ANOVA test, *P* value of Tukey's *post hoc* test between groups were presented within panels. **P < 0.01 vs. LPS group. ^#^ *P* < 0.05, ^##^ *P* < 0.01 vs. LPS+DHQ group. **(G)** Flow cytometeric analysis of intracellular HO-1 in macrophages showed increased HO-1 after DHQ or CC treatment. *P* < 0.0001 in one-way ANOVA test, *P* value of Tukey's *post hoc* test between groups were presented within panels. **P < 0.01 vs. LPS group. ^##^ *P* < 0.01 vs. LPS+DHQ group.

To further clarify the mechanism by which DHQ reduces inflammation, we explored the potential involvement of HO-1. We measured cellular levels of HO-1 protein. As shown in **Figure 3A** (up panel), DHQ increased the HO-1 content of Raw264.7 cells. Also, we further found that this effect was time-dependent, with the most remarkable change observed after 6 h (**Figure 3A** down panel). To corroborate this finding, flow cytometric analysis also indicated that DHQ could significantly strengthen intracellular HO-1 content (0.37 ± 0.03% vs. 18.03 ± 1.30%, *P* = 0.0002) (**Figure 3B**). Quantitative real-time PCR analysis also indicated that LPS challenge induced a nearly 10-fold increase in HO-1 expression (1.00 ± 0.20 vs. 9.09 ± 1.48, p = 0.0082), while DHQ further upregulated HO-1 expression (9.09 ± 1.48 vs. 18.47 ± 2.90, *P* = 0.0456), indicating a potent inducing effect of DHQ on HO-1 in the context of inflammation (**Figure 3C**).

HO-1 is one of the major cytoprotective proteins regulated by Nrf2 activation. Therefore, we measured the effect of DHQ treatment on Nrf2 levels in macrophages to investigate whether Nrf2 was involved in DHQ-induced HO-1 upregulation. We found that DHQ could effectively increase cellular Nrf2 content, which was accompanied by enhanced AMPKα phosphorylation (**Figure 3D**). Immunofluorescence confirmed that DHQ induced nuclear translocation of Nrf2, indicative of activation, which is cancelled by the administration of CC (**Figure 3E**). Moreover, knockdown of HO-1 did not affect the DHQ-induced Nrf2 expression, while the inhibitor of AMPK (CC and si-AMPK) reduced the DHQ-induced Nrf2 expression (**Figures 3E, F** and **Figure S3**) and HO-1 expression (**Figures 3E–G** and **Figure S3**). These results hinted an AMPK/Nrf2/HO-1 axis in the cellular response to DHQ. These results suggest that DHQ could activate HO-1 expression through the AMPK/Nrf2 pathway.

### HO-1 Mediated the Anti-Inflammatory Effect of DHQ

**Figure 4:**
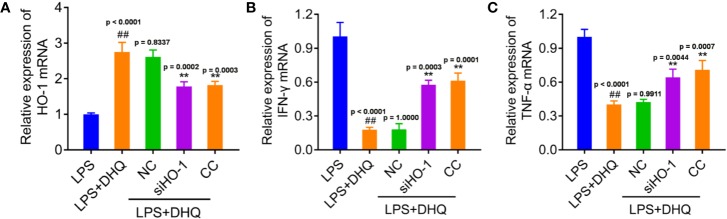
HO-1 mediated the DHQ induced inhibition on the expression of IFN-γ and TNF-α. **(A–C)** RT-PCR analysis of HO-1 mRNA (*P* < 0.0001) **(A)**, IFN-γ mRNA (*P* < 0.0001) **(B)**, and TNFα mRNA (*P* < 0.0001) **(C)** in RAW264.7 cells with indicated treatment. *P* value of Tukey's *post hoc* test between groups were presented within panels. ^##^ *P* < 0.01 vs. LPS group. **P < 0.01 vs. LPS+DHQ group.

In order to verify whether the anti-inflammatory effect of DHQ is mediated by HO-1, we use siRNA to knockdown the expression of HO-1 (**Figure 4A**). As expected, knock down of HO-1 and treatment of CC both recovered the LPS induced IFN-γ and TNFα expression (**Figures 4B, C**), which were the most important pro-inflammatory cytokines. The western blotting analysis showed the expected expression consistent with the mRNA in **Figure S4**. These results suggest the anti-inflammatory effect of DHQ is mediated by AMPK/Nrf2/HO-1 axis.

## Discussion

DHQ was first identified as a plant-derived dihydroflavonol with excellent antioxidant activity. DHQ activates the expression of phase II and antioxidant enzymes *via* the Nrf2−ARE signaling pathway. Several recent studies have described other biological properties of DHQ in several pathophysiological conditions (Liang et al., 2013). Our previous study showed DHQ could activate the Nrf2/HO-1 pathway to ameliorate oxidative stress in concanavalin A-induced liver injury mice. Moreover, DHQ could stimulate BMSCs to differentiate into osteoblasts by suppression of NF-κB signaling pathway (Wang et al., 2017). However, to date, the mechanisms underlying DHQ's anti-inflammatory activities have not been investigated in detail. In this study, we confirmed the AMPK/Nrf2/HO-1 signaling mediated anti-inflammatory effects of DHQ in endotoxemia and provided a basis for clinical use of anti-oxidants in inflammatory diseases.

The potent anti-inflammatory effects of DHQ prompted us to further investigate the molecular mechanisms underpinning its activity in cultured macrophages. In line with the results of our *in vivo* experiments, we found that DHQ could also effectively blunt the inflammatory response of Raw264.7 cells to LPS, reducing expression of inflammatory cytokines. Knockdown of HO-1 cancelled the inhibition of DHQ treatment on LPS induced TNF-α, IFN-γ production, which indicates treatment of DHQ regulates inflammation through Nrf2/HO-1 pathway. Activation of TLR4 by LPS is reported to induce myeloid differentiation factor-88 (MyD88)-dependent pathways (Aderem and Ulevitch, 2000), which then mediate activation of a number of intracellular signaling proteins, including NF-κB, p38 mitogen-activated protein kinase (MAPK). NF-κB and MAPK are tightly associated with the activation of inflammatory cytokines (Zhang L.B. et al., 2017). Our results hint a crosstalk between Nrf2/HO-1 anti-oxidant signaling and TLR4 signaling.

Many electrophilic compounds isolated from plants have been reported to activate the Nrf2-related signaling pathway (Mozahheb et al., 2019). Nrf2 is a member of the basic-leucine zipper transcription factor family that plays important roles in orchestrating cellular responses to oxidative stresses. It is reported that Nrf2 may be activated *via* phosphorylation of serine/threonine residues, and a variety of kinases, including JNK, ERK-1/2, p38 MAPK, and PI3K/AKT, appear to modulate this process (Zhang J. et al., 2017). Our previous study found that AMPKα activation might be a key mediator of the effects of DHQ on Nrf2 activation. Given that AMPK is an upstream sensor and transducer in the signaling link AMPKα activation and nuclear translocation of Nrf2 (Wang et al., 2019; Zhao et al., 2019), targeted gene silence of the kinases by siRNA might provide more insights. Crosstalk between Nrf2 and AMPK signaling pathways was recently reported to be important for the anti-inflammatory effect of berberine in LPS-stimulated macrophages and endotoxin-shocked mice (Mo et al., 2014). This study, in combination with our findings, suggest that uncovering this intersection may illuminate the relationship between energy homeostasis and anti-inflammatory responses and may thus contribute to development of new therapeutic strategies for inflammatory diseases.

Among the molecules regulated by Nrf2, HO-1 has attracted much attention for its versatile roles in maintaining cellular homeostasis. HO-1 is primarily localized in microsomes and is ubiquitously present in mammalian tissue. Under normal physiological conditions, its expression is relatively low. After its activation, HO-1, in addition to influencing the oxidative state, also plays an important protective role in fine-tuning inflammatory response (Ryter and Choi, 2016). Thus potent activation of HO-1 expression by DHQ could explain its anti-inflammatory effects. It is notable that the protective actions of HO-1 are thought to be mediated through by-products of its enzymatic activity, bilirubin and endogenous CO (Poss and Tonegawa, 1997). Endogenous CO inhibits the expression of LPS-induced pro-inflammatory cytokines and increases LPS-induced expression of IL-10 in macrophages, suggesting the anti-inflammatory effect of HO-1 (Otterbein et al., 2000). Conversely, the mice with HO-1 knockout display much stronger inflammatory responses and highly vulnerable to sepsis compared to wide type mice (Poss and Tonegawa, 1997). Considering the antioxidant effect of HO-1, it is possible that deficiency of HO-1 might cause some toxic effect. In our study, knockdown of HO-1 cancelled the inhibition of DHQ treatment on LPS induced TNF-α, IL-6 production, which confirms the anti-inflammatory effect of HO-1.

In summary, our results provide the first experimental evidence of the cellular and molecular mechanisms underpinning the anti-inflammatory properties of DHQ, which extended the lifespan of endotoxemic mice. Our preliminary data suggests that activation of AMPK/Nrf2/HO-1 signaling could play an important role in mediating the protective effects of DHQ during systemic inflammation. Taken together, our results demonstrate the potential value of DHQ as an attenuator of inflammatory disease, and also suggest that activation of AMPK/Nrf2/HO-1 in macrophages may be a useful target for development of anti-inflammatories.

## Data Availability Statement

All datasets generated for this study are included in the article/**Supplementary Material**.

## Ethics Statement

The animal study was reviewed and approved by the Institutional Animal Care and Use Committee (IACUC) of the Guangdong Provincial People's Hospital.

## Author Contributions

LL, YC and HL performed most of the experiments. MZ helped with the in vivo experiments. CC performed the statistical analysis. WX performed the survival analyses. JZ and XF conceived and supervised the study. All authors read and approved the final manuscript.

## Funding

This work was supported by National Natural Science Funds of China (Grant No. 81900285), the National Key Research and Development Program of China (Grant No. 2018YFC1002600), the Science and Technology Planning Project of Guangdong Province China (Grant No. 2017A070701013), Natural Science Foundation of Guangdong Province China (Grant No. 2016A030313792), Science and Technology Program of Guangzhou, China (Grant No. 202002030317) and Guangdong Basic and Applied Basic Research Foundation (Grant No. 2020A1515010242).

## Conflict of Interest

The authors declare that the research was conducted in the absence of any commercial or financial relationships that could be construed as a potential conflict of interest.

## Supplementary Material

Figure S1 Chemical structure of DHQ.

Figure S2 Western blotting analysis showed DHQ pretreatment significantly reduced the levels of TNF-α (*P* < 0.0001), IFN-γ (*P* < 0.0001), IL-10 (*P* < 0.0001) and TLR-4 (*P* = 0.0006) in LPS-challenged groups. Bar graphs illustrate the protein expression of indicated proteins. Values are expressed as mean ± SD. P value of Tukey’s post hoc test between groups were presented within panels. ***P* < 0.01 v.s control group. ^##^ *P* < 0.01 v.s LPS group.

Figure S3 Western blotting analysis of the indicated proteins in the cytoplasm and nucleus of RAW264.7 cells with si-AMPK treatment. Bar graphs illustrate the protein expression of HO-1, p-AMPK, Nrf2. Values are expressed as mean ± SD. *P* < 0.0001 in one-way ANOVA test, *P* value of Tukey’s post hoc test between groups were presented within panels. **P* < 0.05, ***P* < 0.01 v.s LPS group. ^##^ *P* < 0.01 v.s LPS+DHQ group.

Figure S4 Western blotting analysis demonstrated HO-1 mediated the DHQ induced inhibition on the expression of IFN-γ (*P <*0.0001) and TNF-α (*P* =0.003) in RAW264.7 cells with indicated treatment. Bar graphs illustrate the protein expression of indicated proteins. Values are expressed as mean ± SD. *P* value of Tukey's *post hoc* test between groups were presented within panels. ^##^ *P* < 0.01 *v.s* LPS group. ***P* < 0.01 *v.s* LPS+DHQ group.

## Abbreviations

ARRIVE, Animal Research: Reporting of In Vivo Experiments; AMPK, AMP-activated protein kinase; AREs, antioxidant-responsive elements; BSA, bovine serum albumin; CC, Compound C; CO, carbon monoxide; ConA, Concanavalin A; DHQ, Dihydroquercetin; ELISA, Enzyme linked immunosorbent assay; FITC, fluorescein isothiocyanate; HO-1, heme oxygenase-1; IACUC, Institutional Animal Care and Use Committee; IFN-γ, interferon-γ; IL-6; interleukin-6; IL-10, interleukin-10; LPS, lipopolyssacharide; NO, Nitric oxide; NQO1, NAD(P)H quinone oxidoreductase-1; Nrf2, Nuclear factor erythroid 2-related factor 2; PMSF, phenylmethanesulfonyl fluoride; PI, propidium iodide; PVDF, polyvinylidene fluoride; TNF-α, tumor necrosis factor α.
