# Supplementary figures and images for: Dihydroquercetin Activates AMPK/Nrf2/HO-1 Signaling in Macrophages and Attenuates Inflammation in LPS-Induced Endotoxemic Mice
Source: Front Pharmacol. 2020 May 19;11:662. doi: 10.3389/fphar.2020.00662 (PMC7248193; doi:10.3389/fphar.2020.00662)

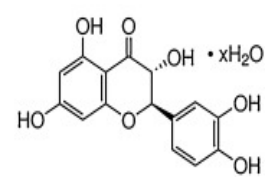

Supplement: Figure S1 — Chemical structure of DHQ. [file Image_1.tif]

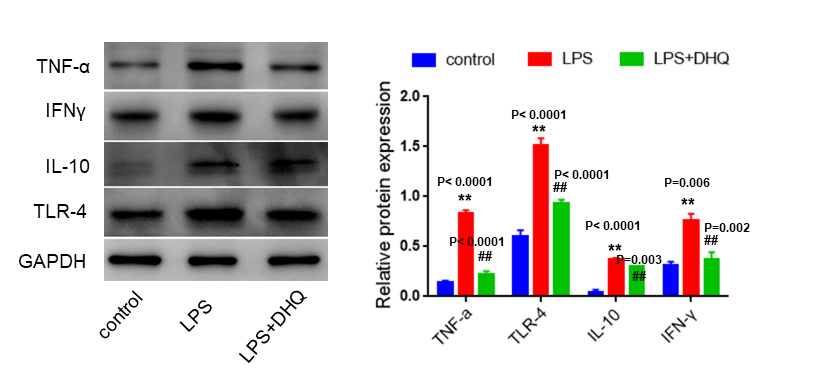

Supplement: Figure S2 — Western blotting analysis showed DHQ pretreatment significantly reduced the levels of TNF-α (P < 0.0001), IFN-γ (P < 0.0001), IL-10 (P < 0.0001) and TLR-4 (P = 0.0006) in LPS-challenged groups. Bar graphs illustrate the protein expression of indicated proteins. Values are expressed as mean ± SD. P value of Tukey’s post hoc test between groups were presented within panels. **P < 0.01 v.s control group. ## P < 0.01 v.s LPS group. [file Image_2.tif]

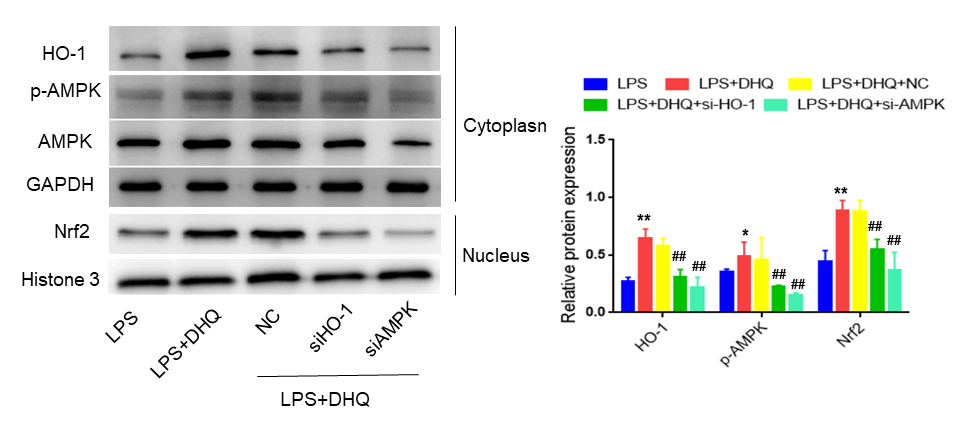

Supplement: Figure S3 — Western blotting analysis of the indicated proteins in the cytoplasm and nucleus of RAW264.7 cells with si-AMPK treatment. Bar graphs illustrate the protein expression of HO-1, p-AMPK, Nrf2. Values are expressed as mean ± SD. P < 0.0001 in one-way ANOVA test, P value of Tukey’s post hoc test between groups were presented within panels. *P < 0.05, **P < 0.01 v.s LPS group. ## P < 0.01 v.s LPS+DHQ group. [file Image_3.tif]

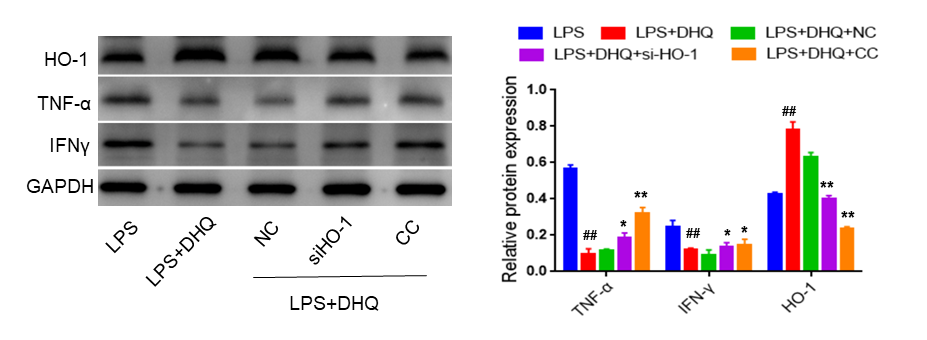

Supplement: Figure S4 — Western blotting analysis demonstrated HO-1 mediated the DHQ induced inhibition on the expression of IFN-γ (P <0.0001) and TNF-α (P =0.003) in RAW264.7 cells with indicated treatment. Bar graphs illustrate the protein expression of indicated proteins. Values are expressed as mean ± SD. P value of Tukey's post hoc test between groups were presented within panels. ## P < 0.01 v.s LPS group. **P < 0.01 v.s LPS+DHQ group. [file Image_4.tif]
